# Polypus Uteri

**Published:** 1842-02

**Authors:** 


### POLYPUS UTERI.

Doctors Page and Geoghegan, of Henry county, Ky., have sent us the notes of a case of polypus uteri, for three years mistaken by different physicians, for *prolapsus U. procidentia U.* and *inversio U.* Throughout the whole of that period, the patient, a negress, who had borne one child, *suffered extreme agony* in the vagina and uterus. The application of a metallic ligature, by means of a double canula, was soon followed by an abominable exhalation from the vagina, which continued till the eleventh day, when the tumor was detached. Its weight was sixteen ounces. We hope they will send it to the Obstetrical Department of our pathological museum.

